# Being a scientific editor at *Brain Communications*

**DOI:** 10.1093/braincomms/fcad181

**Published:** 2023-07-01

**Authors:** Manuela Marescotti

**Affiliations:** Edinburgh, UK

## Abstract

Our Scientific Editor discusses her role at *Brain Communications*.

## Graphical Abstract

**Graphical abstract:**
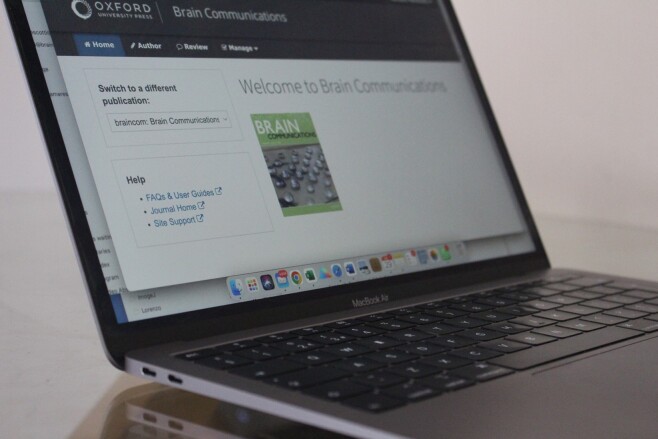

Welcome to Volume 5, Issue 4 of *Brain Communications*. In this editorial, I would like to shed light on the role of the Scientific Editor within our journal. I’m Dr Manuela Marescotti and I joined *Brain Communications* in 2019 in this role, being a neuroscientist by training and after spending more than 10 years as a bench-scientist.

Publishing peer-reviewed articles is at the heart of scientists’ careers where the number of published papers in a CV is often used as a proxy for productivity.

Within this scenario, scientists are usually familiar with what they see of this system: their involvement as either author or reviewer of these documents; but the backstage workings of the journals publishing their papers are often a mystery. Here, I will give you insights about the job of the Scientific Editor at *Brain Communications*, aiming to provide you with a better understanding of the peer review process, to improve your experience in submitting papers, and to highlight a stimulating career option that might interest some readers.

First, I’d like you to picture a peer-reviewed journal as a pipeline and with a constant flow of articles both coming in (submitted) and out (sent back to the authors with a decision).^1^ Within this context, the editors play a crucial role in collaboration with the reviewers to carefully check the content of the manuscripts before sharing it with the scientific community. The different editorial roles take care of different aspects.

When a new paper is submitted to our journal, the Editor-in-Chief and Associate Editors check its suitability for *Brain Communications.* At this stage, if the manuscript is not in scope for our journal or has major flaws that we think make it not in line with our standards of rigour and reproducibility, it is rejected. If it is not immediately rejected, members of the editorial team search for scientists (reviewers) with expertise as close as possible to the topic of the paper to examine it carefully. Next, the reviewers give their feedback and the Associate Editors make their recommendations based on those comments. Then, I or one of our Assistant Scientific Editors come into play and check if the data and analyses conform to the journal’s standard, since our journal aims to ensure a ‘high standard of rigour and transparency’ of data. This quality check is done at every round of revisions for each manuscript. We check things like reporting of the sex of subjects, appropriateness of statistical analyses, and that figures are of high quality.

In addition, I commission commentaries about articles published in *Brain Communications*, based on topics that may currently be a matter of debate, and, I often contribute to researching suitable reviewers to evaluate the papers the journal receives. Last but not least, I work towards promotion of transparency and career development through two initiatives that involve training of early career scientists as reviewers (Reviewer Academy), and increasing the transparency of *Brain Communications’ modus operandi* (Observers Programme).

Overall, I feel this role is the natural development of my scientific career, as it requires both a critical and curious attitude that I developed while being a lab-based scientist. Moreover, reading articles covering different areas of neuroscience, different methodologies, and data analyses, I have the opportunity to keep myself updated and to learn every day.

If you would like to know more about how *Brain Communications* works and our journal’s initiatives, please, visit our website’s dedicated pages https://academic.oup.com/braincomms/pages/observers and https://academic.oup.com/braincomms/pages/reviewer-academy and follow our Twitter account @braincomms to be kept updated about active recruitment times for these programmes!

The cover of this issue comes from Professor Deb Pal (King’s College London) and shows a black swan to exemplify a fallacy of defective induction: ‘if all swans are white, black swans cannot be swans’, a conclusion that can result from weak assumptions.
